# Surveillance of Occupational Exposure to Volatile Organic Compounds at Gas Stations: A Scoping Review Protocol

**DOI:** 10.3390/ijerph21050518

**Published:** 2024-04-23

**Authors:** Tatiana de Medeiros Carvalho Mendes, Juliana Pontes Soares, Pétala Tuani Cândido de Oliveira Salvador, Janete Lima de Castro

**Affiliations:** 1 Postgraduate in Collective Health, Federal University of Rio Grande do Norte, Natal 59056-000, Brazil; janete.castro@ufrn.br; 2 Insikiran Institute of Indigenous Higher Education, Federal University of Roraima, Boa Vista 69310-000, Brazil; juliana.pontes@ufrr.br; 3 School of Health, Federal University of Rio Grande do Norte, Natal 59078-900, Brazil; petala.salvador@ufrn.br

**Keywords:** gas station worker, public health surveillance, monitoring, occupational exposure, gasoline, benzene, toluene, ethylbenzene, xylene

## Abstract

Health surveillance guides public policies, allows for the monitoring of occupational exposures that may cause health risks, and can prevent work-related diseases. The scoping review protocol herein is designed to map studies on the surveillance of occupational exposure to volatile organic compounds (VOCs) in gas stations and identify the governmental agencies and public health measures in different countries. This review protocol is based on the Joanna Briggs Institute manual and guided by the PRISMA Extension for Scoping Reviews. It includes research articles, theses, dissertations, and official documents on surveillance measures for occupational exposure to VOCs (i.e., benzene, ethylbenzene, toluene, and xylene) in gas stations from different countries. All languages and publication dates will be considered, and a spreadsheet will be used to extract and analyze qualitative and quantitative data. The final version will present the main surveillance measures implemented, responsible entities, results, challenges, limitations, and potential gaps in gas stations.

## 1. Introduction

Health surveillance guides public policies, health information and interventions, improvements in communication, and the assessment of research investments. It also helps to prevent work-related diseases through the monitoring of occupational exposures that may cause health risks [1]. According to a study including data from 195 countries [2], the global cancer burden caused by occupational exposure to carcinogens is increasing, highlighting the need to monitor such exposure.

Occupational exposure monitoring refers to the continuous and systematic assessment of concentrations of chemical, biological, or physical agents present in the workplace environment and the evaluation of workers’ exposure to these agents. This exposure poses various health risks, which vary depending on the agents or conditions present in the workplace [3,4]. These risks can trigger various health problems, such as infectious diseases, mental and behavioral disorders, respiratory and circulatory issues, digestive diseases, and cancers, among others [5].

Although the International Labour Organization conventions 155 and 161 provide legal frameworks for occupational safety and health protection (i.e., in the workplace) [6,7], many workers still fall ill due to poor working conditions worldwide. Gas station workers are exposed to multiple health risks. Thus, workplace and occupational management measures are important to prevent adverse environmental impacts on surrounding populations and the health of workers [8].

Gasoline, a globally distributed fuel in gas stations, consists of light liquid fractions of petroleum known as gasoline range organics (GROs). GROs contain chains of six to ten carbons and many aliphatic and aromatic hydrocarbons, such as benzene, toluene, ethylbenzene, and xylenes (BTEX) [9].

Benzene is highly relevant toxicologically due to its high health risk, having been classified as a Group 1 carcinogen by the International Agency for Research on Cancer (IARC) with no established safe exposure limit [10]. The literature shows that exposure to benzene, even below the occupational limit (1 ppm), poses health risks to workers [11]. Long-term benzene exposure causes hematological and genotoxic damage [12] and is associated with high risk of cancer [13], such as chronic myeloid leukemia and lung cancer [14]. Moreover, although toluene, ethylbenzene, and xylene have not shown risks for cancer [15], workers chronically exposed to BTEX have presented adverse effects such as fatigue, headache, dizziness, nasal congestion, runny nose, chest pain, epistaxis, anemia, muscle weakness, drowsiness, tight chest, tachycardia, petechiae, and unconsciousness [16].

Considering that occupational exposure causes health risks ranging from potentially reversible alterations to mortality, the assessment of occupational exposure to chemical compounds is important with respect to the health status of workers. In this context, the exposure of gas station workers to volatile organic compounds (VOCs)—especially benzene—is concerning [8].

Surveillance measures focused on occupational health in gas stations involve systematic assessment, improvement of workplaces and working conditions, and monitoring of the health status of workers. They may also identify risks and the need for preventive and health protection measures [17].

Considering the importance of surveillance measures concerning occupational exposure in gas stations, a preliminary search in electronic databases was conducted in August 2023. The aim was to systematize evidence, identify the measures implemented, and guide policies for the health protection of workers. The electronic databases used were Joanna Briggs Institute (JBI), The Cochrane Library, PROSPERO, Open Science Framework (OSF), and Medline. This search identified two systematic reviews assessing the health risks of workers exposed to VOCs in gas stations [18,19] and three ongoing review protocols restricted to exposure assessment and effects on the health status of workers [20,21,22]. However, an expanded scope beyond the surveillance of the health status of workers—including intervention studies in workplaces focused on working conditions and surveillance measures of occupational exposure to BTEX conducted by government agencies and public health services—is yet to be found.

Thus, this study proposes a scoping review protocol to map studies on the surveillance of occupational exposure to VOCs in gas stations, aiming to identify the surveillance measures developed by governmental agencies and public health services in different countries.

## 2. Materials and Methods

This scoping review protocol was designed following the JBI manual and the theoretical framework proposed by Arskey and O’Malley [23], updated by Levac, Colquhoun, and O’Brien [24] and Peters et al. [25]. In addition, the Preferred Reporting Items for Systematic Reviews and Meta-Analyses Extension for Scoping Reviews (PRISMA-ScR) guided its development [26]. The protocol was developed and registered on OSF and can be accessed through the following link: http://doi.org/10.17605/OSF.IO/6JK5Z (accessed on 16 January 2024).

**Figure 1:**
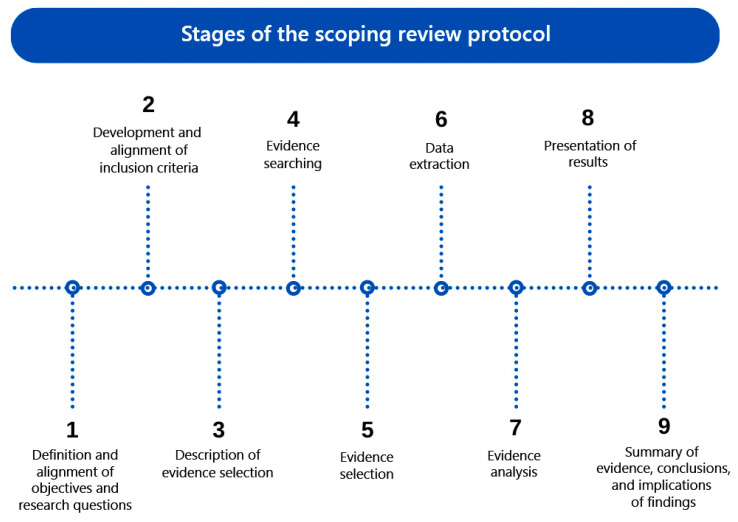
Stages of the scoping review protocol.

The scoping review protocol follows nine steps, as shown in Figure 1.

### 2.1. Stage One: Definition and Alignment of Objectives and Research Questions

The population, concept, and context (PCC) framework was used to define the research question. The PCC framework enables the mapping of various information, identification of potential knowledge gaps, presentation of key concepts, broad quantification of aspects of interest, and determination of practices and evidence within a specific thematic area [27].

For this review, we establish P as workers, C as the surveillance of occupational exposure to VOCs in gas stations, and C as gas stations (Table 1).

Thus, the research questions are as follows: What studies on surveillance of occupational exposure to VOCs in gas stations in different countries have been published? What surveillance measures regarding occupational exposure to VOCs at gas stations have been conducted by governmental agencies or public health services?

### 2.2. Stage Two: Development and Alignment of Inclusion Criteria

We will include full-length research articles, theses, dissertations, and official documents on surveillance measures for occupational exposure to BTEX in gas stations in different countries. All language and publication dates will be considered, while duplicate studies, review articles, letters, editorials, and opinion pieces will be excluded.

### 2.3. Stage Three: Description of Evidence Selection

Initial searches were conducted in English on PubMed using Medical Subject Headings (MeSH) and in Portuguese using Health Sciences Descriptors (DeCS) to identify the main descriptors, synonyms, and keywords related to the studied theme.

Then, two librarians refined the strategy, using four controlled health vocabularies (DeCS, MeSH, Cinahl Thesaurus, and EMTREE) to obtain more studies. Moreover, the use of natural language enhanced sensitivity and expanded the results (Appendix A) [30,31].

The search strategy was developed using the extraction, conversion, combination, construction, and utilization model [30]. This model allows for the development of a highly sensitive strategy by following complementary steps. Table 2 shows the conversion of mnemonic elements into main keywords.

Using this highly sensitive search strategy, data will be collected from Scopus, Web of Science, Medline/PubMed, Embase, Cinahl, Engineering Village, and Lilacs. The grey literature search will include Google Scholar, Digital Library of Theses and Dissertations, Catalog of Theses & Dissertations—CAPES, Open Access Theses and Dissertations, ProQuest Dissertations & Theses Global, and Networked Digital Library of Theses and Dissertations.

Additional sources from the references of selected studies will be further searched. If needed, the authors will be contacted for further information.

### 2.4. Stage Four: Evidence Searching

For data collection, an appropriate strategy for each database will be employed. The studies found will be transferred to Rayyan software (http://rayyan.qcri.org, accessed on 5 April 2024) (Qatar Foundation, Doha, Qatar) [32] for the removal of duplicates and classification of the studies based on their titles and abstracts.

The reviewers will perform a pilot test in which 25 studies will be randomly assessed based on their title and abstract to verify the eligibility criteria. A minimum agreement of 75% must be achieved before collecting the data independently [23].

After the pilot test, two independent reviewers will analyze the titles and abstracts using the Rayyan software (T.d.M.C.M and J.P.S). Discrepancies between the reviewers will be resolved through discussion and, if needed, a third reviewer (P.T.C.d.O.S) will be consulted.

### 2.5. Stage Five: Evidence Selection

The selected studies will be fully retrieved, and two independent reviewers (T.d.M.C.M and J.P.S) will conduct full-text reviews. Reasons for exclusion will be recorded and reported.

The selection details for peer-reviewed and grey literature should be presented as a flowchart, following PRISMA-ScR guidelines [26]. At this stage, the reviewers will perform a new search across all databases to check for further studies for possible inclusion.

### 2.6. Stage Six: Data Extraction

Two independent reviewers will extract the data highlighted in Table 3, using a form built in Microsoft Excel^®^.

### 2.7. Stage Seven: Evidence Analysis

Quantitative data will be analyzed using descriptive statistics and presented in absolute or relative frequency. Qualitative analysis will involve identifying meanings and patterns to address the research questions according to the thematic content analysis proposed by Bardin, employing preanalysis, material exploration, result treatment, inference, and interpretation [33]. The data will be analyzed considering the diverse realities among nations.

### 2.8. Stage Eight: Presentation of Results

The PRISMA-ScR guidelines will guide the final report [26]. The results should be discussed based on the relevant literature and presented in flowcharts, graphs, or tables.

The preliminary results will be shared via email with five experts in the field of surveillance of occupational health and researchers. This step will improve the strength of the review, as well as favoring socialization and knowledge sharing. Additionally, it will enable further knowledge expansion using new evidence not identified in the review [25].

An electronic form with the summarized results and informed consent will be emailed to the experts. Stakeholders will not be identified, and the authors will request an analysis of the results and potential new fields or evidence. This stage was approved on 6 April 2023 by the research ethics committee of Onofre Lopes University Hospital (no. 5.989.389 and CAAE no. 67143923.4.0000.5292).

### 2.9. Stage Nine: Summary of Evidence, Conclusions, and Findings Implications

In this stage, the results will be summarized according to the objective, allowing knowledge gaps to be highlighted and indicating future studies in the addressed theme.

## 3. Discussion

The proposed protocol will guide a scoping review to map studies on the surveillance of occupational exposure of workers to VOCs in gas stations, regarding their health status, surveillance measures implemented in workplaces, and working conditions.

Occupation and working conditions are social determinants of health, which affect health inequalities [34]. In this context, developing surveillance measures for exposure to VOCs in gas stations impacts workers, surrounding populations, and the environment [35]; as such, understanding these measures and successful experiences in the area is crucial.

A research team of experts in scoping reviews and occupational health developed the protocol. Two librarians developed a highly sensitive search strategy without date or language limitations, aiming for broad access to the existing literature. All information obtained in the scoping review stages enhances transparency, thus supporting methodological replication and minimizing biases and data duplication, according to the principles of open science.

The results are to be reported following the PRISMA-ScR guidelines [25] and summarized to understand the surveillance measures of occupational exposure to VOCs in gas stations developed in diverse contexts. Additionally, responsible entities for the measures, impacts, limitations, challenges, and potential gaps may guide future research. Moreover, the findings are expected to benefit employers, managers, workers, and health policymakers.

As a limitation, searching the grey literature of some countries is infeasible and cannot be performed. However, comprehensive and essential databases incorporating other sources of grey literature can be selected. Furthermore, there is the possibility of encountering a large amount of heterogeneous data, considering the diverse realities of various countries.

## 4. Conclusions

The proposed scoping review protocol allows for the methodological identification of studies on the surveillance of occupational exposure to VOCs in gas stations and the surveillance measures developed by governmental agencies and public health services in different countries.

The results should be published in peer-reviewed open-access journals to disseminate knowledge on surveillance measures developed for gas station workers. Identifying which surveillance methods have been used in various countries to monitor the concentrations of chemical compounds in work environments, in inspections of physical conditions at gas stations, as well as while workers’ health was monitored, will provide important information for the prevention of the onset of illness and improvements in health and safety conditions in work environments, in addition to potentially guiding policies aimed at protecting worker health.

Changes to this protocol will be reported in the final version, including dates and justifications.

## Figures and Tables

**Table 1:** Definition of concepts used in the review.

Mnemonic Elements	Concept		Definition
P	Workers		All employed individuals [6].
C	Surveillance of occupational exposure to VOCs such as BTEX found in gas stations.	Surveillance in gas stations	Systematic assessment, improvement of workplaces and working conditions, and monitoring of the health status of workers in gas stations [17].
		Health surveillance of workers	Continuous and systematic measures to understand, research, and analyze the determining and conditioning factors of health risks from workplaces and working conditions. Its purpose is to plan, execute, and assess interventions on these aspects, aiming to eliminate or control them [28].
		VOCs	The main VOCs in fuels that may cause health risks include BTEX [15].
C	Gas stations		A workplace that commercializes fuels and supplies fuel tanks for motor vehicles [29].

**Table 2:** Conversion of adopted mnemonic elements.

Mnemonic Elements	Extraction	Conversion
Population	Gas station workers	Worker Gas station worker
Concept	Surveillance measures for exposure of workers to BTEX ^1^	Public Health Surveillance monitoring Occupational exposure to BTEX ^1^
Context	Gas stations	Gasoline Gas stations

^1^ BTEX: benzene, toluene, ethylbenzene, and xylene.

**Table 3:** Instruments for data extraction.

Variable	Standardization
Type of material	Article, dissertation, thesis, or official documents.
Year of publication	The year when the study was published.
Publication context	The location where the study was conducted.
Author formation	The education level of the first author, as indicated in the study.
Objective	Detail the objective of the study.
Type of study	Detail the type of research described by the author(s).
Responsible entities for the surveillance measures	List those responsible for the surveillance measures (e.g., researchers, government agencies, companies, or official institutions).
Study population	Categories of workers benefiting from the surveillance measures (e.g., attendant, administrative assistant, manager, and janitor).
Hours worked per day/exposure time	Detail how many hours workers are exposed per day to the chemicals in the workplace.
Measures developed	To map developed measures.
Technological tools for preventing the spread of BTEX ^1^ in the air.	Utilization of technological tools that can help prevent the spread of BTEX ^1^ in the air.
Results/impacts	Cite the main results/impacts of the studies.
Limitations/challenges	List the potential challenges/limitations reported.

^1^ BTEX: benzene, toluene, ethylbenzene, and xylene.

## Data Availability

Not applicable.

## Appendix A

**Table A1:** Complete search strategy used for Medline/PubMed.

Search	Terms	Retrieved Studies (29 December 2023)
#1	worker[Title/Abstract] OR laborer[Title/Abstract] OR labourer[Title/Abstract] OR “gas station worker”[Title/Abstract] OR “gas station workers”[Title/Abstract] OR “gas station attendant”[Title/Abstract] OR “gas station attendants”[Title/Abstract] OR “gas station operator”[Title/Abstract] OR “gas station operators”[Title/Abstract] OR “gas station employee”[Title/Abstract] OR “fuel station workers”[Title/Abstract] OR “fuel station attendant”[Title/Abstract] OR “fuel station attendants”[Title/Abstract] OR “fuel station operator”[Title/Abstract] OR “fuel station operators”[Title/Abstract] OR “fuel station employee”[Title/Abstract] OR “workers of gas station”[Title/Abstract] OR “workers of fuel station”[Title/Abstract] OR “petrol station attendant”[Title/Abstract] OR “petrol station attendants”[Title/Abstract] OR “petrol station worker”[Title/Abstract] OR “petrol station workers”[Title/Abstract] OR “petrol station operators”[Title/Abstract] OR “petrol station operator”[Title/Abstract] OR “occupational groups”[Title/Abstract] OR employees[Title/Abstract] OR personnel[Title/Abstract] OR workers[Title/Abstract] OR “occupational group”[Title/Abstract] OR employee[Title/Abstract] OR workplace[Title/Abstract] OR workplaces[Title/Abstract] OR “work location”[Title/Abstract] OR “work locations”[Title/Abstract] OR “work-site”[Title/Abstract] OR “work site”[Title/Abstract] OR “work-sites”[Title/Abstract] OR “work place”[Title/Abstract] OR “work places”[Title/Abstract] OR “job site”[Title/Abstract] OR “job sites”[Title/Abstract] OR worksite[Title/Abstract] OR worksites[Title/Abstract]	403,749
#2	monitoring[Title/Abstract] OR surveillance[Title/Abstract] OR (biomarkers[Title/Abstract] “environmental monitoring”[Title/Abstract] OR “monitoring biological”[Title/Abstract] OR “environment monitoring”[Title/Abstract] OR “environmental parameter monitoring”[Title/Abstract] OR “genotoxicity test”[Title/Abstract] OR “manage the health risks”[Title/Abstract] OR “manage the health risks”[Title/Abstract] OR “risk assessment”[Title/Abstract] “environmental surveillance”[Title/Abstract] OR “public health surveillance”[Title/Abstract] OR “occupational health surveillance”[Title/Abstract] OR “health surveillance”[Title/Abstract] OR “occupational exposure”[Title/Abstract] OR “occupational exposures”[Title/Abstract] OR exposure[Title/Abstract] OR “occupational hazard”[Title/Abstract] OR “occupational diseases”[Title/Abstract] OR “occupational disease”[Title/Abstract] OR “occupational disorder”[Title/Abstract] OR “occupational dysfunction”[Title/Abstract] OR “occupational illness”[Title/Abstract] OR “occupational illnesse”[Title/Abstract] OR “occupational illnesses”[Title/Abstract] OR “professional disease”[Title/Abstract] OR “occupational health”[Title/Abstract] OR “industrial hygiene”[Title/Abstract] OR “industrial health”[Title/Abstract] OR “occupational safety”[Title/Abstract] OR “employee health”[Title/Abstract] OR “professional health”[Title/Abstract] OR “surveillance of the workers health”[Title/Abstract] OR “occupational health policy”[Title/Abstract]	1,080,027
#3	benzene[Title/Abstract] OR benzol[Title/Abstract] OR benzole[Title/Abstract] OR toluene[Title/Abstract] OR methacide[Title/Abstract] OR methylbenzene[Title/Abstract] OR phenylmethane[Title/Abstract] OR “toluene isomer”[Title/Abstract] OR toluol[Title/Abstract] OR tolylene[Title/Abstract] OR ethylbenzene[Title/Abstract] OR “ethyl benzene”[Title/Abstract] OR xylenes[Title/Abstract] OR dimethylbenzene[Title/Abstract] OR xylene[Title/Abstract] OR xylol[Title/Abstract] OR BTX[Title/Abstract] OR BTEX[Title/Abstract]	72,013
#4	gasoline[Title/Abstract] OR benzine[Title/Abstract] OR “benzine vapor”[Title/Abstract] OR “benzine vapour”[Title/Abstract] OR petrol[Title/Abstract] OR “petrol poisoning”[Title/Abstract] OR “diesel fuel”[Title/Abstract] OR “diesel oil”[Title/Abstract] OR “gas station”[Title/Abstract] OR “fuel station”[Title/Abstract] OR “gas stations”[Title/Abstract] OR “fuel stations”[Title/Abstract] OR “petrol station”[Title/Abstract] OR “petrol stations”[Title/Abstract] OR “filling station”[Title/Abstract]	9711
	#1 AND #2 AND #3 AND #4	214
#5	(((((((((((occupational groups[MeSH Terms]) OR (workplace[MeSH Terms])) AND (public health surveillance[MeSH Terms])) OR (environmental monitoring[MeSH Terms])) OR (biological monitoring[MeSH Terms])) OR (occupational exposure[MeSH Terms])) OR (occupational diseases[MeSH Terms])) AND (benzene[MeSH Terms])) OR (toluene[MeSH Terms])) OR (ethylbenzene[Supplementary Concept])) OR (xylenes[MeSH Terms])) AND (gasoline[MeSH Terms])	205
	#1 AND #2 AND #3 AND #4 OR #5	369

Note: # refers to each part of the search strategy presented in the rows of the table. In the end, the crossing was performed between them for the search.
